# The Right Kidney—Disquieting Factor in the Diagnosis of Acute Intra-Abdominal Conditions

**Published:** 1913-02

**Authors:** W. L. Peple

**Affiliations:** Richmond, Va.


### THE RIGHT KIDNEY—DISQUIETING FACTOR IN THE DIAGNOSIS OF ACUTE INTRA-ABDOMINAL CONDITIONS

By W. L. Peple, Richmond, Va.

If the thumb of the right hand is laid along the lower right costal margin with the tip of the little finger resting on the anterior superior iliac spine, the palm and extended fingers will cover the Balkans of Surgery, a hotbed of disaffection and rebellion, a veritable zone of discontent.

Here lie the four allies, the gall-bladder, kidney, appendix and fallopian tube. It is not my purpose to attempt to fix individual responsibility for acts of each or all, for all, save one, are open to fronted attacks and a clever blow, well timed and skillfully delivered may well strike three, but the fourth must be taken in the rear, and is far oftener vulnerable to diplomacy than to open war. In other words, it is not my intent to enter into a discussion of the differential diagnosis of the diseases of these four organs, but to call attention to certain disorders of the kidney which simulate at times so closely diseases of the other three.

This is especially important because abdominal incision, even if made over the wrong organ, will do little harm and guide us to the right one. But an abdominal incision will not aid us in operating upon a kidney or ureter in need of surgery; and an unnecessary traumatism and a general anesthetic can only make worse a purely medical disease of the kidney.

I do not wish to minimize the importance of a careful differential diagnosis of the diseases of the right abdomen, but wish to impress the greater importance of fixing the responsibility upon the kidney when it is at fault, and of eliminating it as a foctor when innocent.

*Acute unilateral nephritis.*—The term "nephritis" is so inseparably linked with the name of Bright that when we hear it we instinctively picture it either as a local expression of a systematic disorder or the occompaninment of one of the acute, infectious diseases.

The term "pyelitis" carries with the old text-book picture of chills, sweats, high fever, pain in the back, and urine loaded with visible pus—terminal condition bearing about the same relation to early infection as does general peritonitis to the intact appendix with an inflamed mucosa.

The picture of a blood-borne infection affecting but one kidney is to some of us novel, if not quite unique. Ordinarily, the surgeon does not see acute, abdominal infections early. When he does, time enough has usually elapsed for definite symptoms to have emerged from the chaos overshadowed by pain, and his task is made much easier.

Given a case of severe, right-sided abdominal pain with elevation of temperature acceleration of pulse, vomiting and local tenderness, what should be our method of procedure? First, elimination of the tubes by careful vaginal examination. This should be a routine measure and it is one far too frequently neglected. If there is one point which surgical opinion has crystallized, it is the nonoperative treatment of acute aubal infections. The next step is the elimination of the kidney, and, this, to my mind, is one of the most difficult and disquieting procedures in surgical diagnosis.

If there is a second point on which surgeons have agreed it is the early operative treatment of acute infections of the appendix. The differential diagnosis is of paramount importance. If the case is one of acute, unilateral nephritis or pyelitis, there is not only no need for appendectomy, but the traumatism and the anesthetic will do the inflamed kidney positive harm.

As I have seen practically nothing written on this interesting condition, I must give the symptoms as I have observed them at the bedside, hoping that in the discussion, the experience of others will add definiteness to a rather meager picture.

The pain is intense and of a violent, boring character. It is far more prone to exacerbations and remissions than pain from the appendix. It seldom, if ever, begins as a general abdominal pain, but starts over the appendix. It is, has been, or at some time will be, over the kidney region of the back, though not so severe as anteriorly. The point of greatest tenderness anteriorly has been below and internal to McBurney's point. Why, I do not know. Tenderness can almost be elicited over the kidney also by pressure from behind or on bimanual palpation of the organ. The facial expression has nothing characteristic and is never anxious. I have not observed muscular rigidity or tympany. The temperature is a variable quantity, being very high in some cases and but slightly elevated in others. The pulse keeps pace with the temperature, and has nothing characteristic about it.

The crucial test is the microscopic examination of the urine. To avoid possible sources of error, I would emphasize the necessity of excluding gonorrhoea in the male by inspection as well as questioning, and by the invariable use of the catheter in the female; for an uncatheterized specimen in the female is worse than valueless—it may be positively misleading. Furthermore, the examination should be made by one skilled in this kind of work.

*Urinary Findings.*—In the early stage, there will be found a few red blood-cells, a few leucocytes, blood casts, fibrin casts, and epithelium from the kidney tubules. The blood and blood-casts vary in wide limits according to the severity of the case. As time passes, the blood diminishes and the pus-cells increase in number. When the pelvis of the kidney is most affected, the blood-casts are absent and pus-cells more abundant.

The following case-histories will illustrate the condition described:

Case 1. Male; single; white; age, 38; no history of a previous similar attack. Following a rather free partaking of alcoholics, he was seized with violent, right abdominal pain, high temperature, rapid pulse, and vomiting. He was seen about six hours after the attack began. There was marked tenderness a little below and internal to McBurney's point, but no rigidity. The pain had also been in the kidney region behind. Dimanual pressure over the kidney revealed a very tender organ.

*Diagnosis.*—Acute, right, unilateral nephritis. Having observed similar cases, I predicted the urinary findings for three days, and was not a little pleased to have them subsequently verified: 1st day: a few blood-cells, red and white; a few blood-casts and kidney epithelium. 2nd day: less blood and more pus-cells. 3rd day: a much freer showing of pus.

Case II. Female; white; married; age 20; menses normal; never pregnant. She came with a diagnosis of acute appendicitis. One year before she had an attack of pain over the ureters, more on the left than on the right side, accompanied by bloody urine lasting several days; then followed an interval of several months without further trouble. She then suffered with a pain in the back and over the ureters, and was on and off of the bed for about five weeks with paroxysmal pains at intervals of several days. At times there was blood in the urine as small clots, but it was not cloudy. She now developed chills which were without any regular periodicity. These gradually disappeared and she was up and about again. While walking from a neighbor's, she was seized with sudden pain over the appendix so severe that she reached home with difficulty. The pain continuing, she was brought to the hospital, the diagnosis being acute appendicitis. The temperature was normal, the pulse rapid, and there was no rigidity or distension. Marked tenderness a little below and internal to McBurney's point and to a less degree over the kidney front and rear, was present. Vaginal examination was negative, Urinalysis showed a small amount of blood and pus, renal epithelium and blood-casts.

*Diagnosis.*—Diffuse nephritis and pyelitis.

In a few days, there was a flocculent cloud of pus in the urine. The temperature varied from 100 to 103 degrees. The pain continued paroxysmally; the tenderness over the appendix persisted, but subsided with the rest of the symptoms. A radiograph was now taken and proved negative. The appendix, which was slightly, if at all, diseased, was then removed.

Case III. White; male; single; aged 24; gave rather a unique history in that his appendix, chronically inflamed and badly adherent, had been removed several years previously. After six weeks confinement to bed because of a fractured thigh, while convalescent he was seized with violent pain in the right abdomen. The pain was rather high, and as the appendix had been removed, the case was diagnosed as acute cholecystitis. Microscopic examination showed a large number of blood-and fibrin-casts, which fixed the responsibility on the kidney.

Case IV. White; female; single; stenographer; aged 22, gave a history of several attacks slight in character and short in duration. The present attack was far more severe than any other. The pain was very severe paroxysmal, beginning in the lumbar region and localizing over the appendix. Rigid and tympany were absent. There was tenderness over the appendix as well as in the lumbar region.

*Diagnosis.*—Acute, unilateral nephritis, confirmed by microscopic examination.

The patient states that in the past four years she has had several attacks which have yielded readily to urinary antiseptics.

Case V. White; female; single; aged 22; gave a history of ovarian neuralgia two years before the present trouble. A temperature of 100 degrees was preceded by three days of pain over the appendix. The diagnosis of appendicitis was made; and I was called in to operate when all preparations had been made. Hasty examination revealed a very tender spot over the appendix. A normal appendix was removed. The temperature and pain persisted; and a few days later the urine showed a heavy, flocculent cloud of pus. She had a protracted convalescence, but made a good recovery. This was, to my mind, a case of pyelitis from the first, that a more painstaking examination would have revealed.

Case VI. Colored; male; aged 45; farmer; was suddenly seized with violent pain in the right lumbar region, radiating anteriorly. On palpatation, the kidney was found to be quite tender, front and rear. A tentative diagnosis of stone was made. Urinalysis showed a few leucocytes and blood-cells, radiograph was negative.

*Diagnosis.*—Acute, right, unilateral nephritis.

Under urinary antiseptics, recovery was rapid.

*Stone in the Kidney and Ureter.*—If one doubts the confusion of symptoms of stone with these of appendicitis, let him look over a series of cases of stone and see what a goodly percentage of them have scars over McBurney's point. We have all had the experience of opening the abdomen and finding an appendix the pathology of which would in no way warrant the severity of symptoms represented. I believe that a careful subsequent analysis of the urine and a radiograph taken by a competent operator will do more to aid us in arriving at a correct solution of the trouble than a second operation and a search for adhesions and kink of Lane.

The symptoms of stone in the right kidney or ureter are, briefly, sudden, usually violent, pain, never starting as a general abdominal pain, but beginning in the lumbar region and radiating to the appendicular region, or vice versa. I have rarely observed the reflex pain in the head of the penis, so frequently noted in print. Fever is not a usual accompaniment. The whole general picture is one less of surgical urgency than an intra-abdominal inflammation.

Careful microscopic examination of the urine will almost invariably show blood and epithelium from the kidney, pelvis or ureter; and indicates the need of a confirmatory radiograph. I am indebted to two of my colleagues for invaluable assistance in the foregoing and following cases: Dr. E. Guy Hopkins, for the pathologic; and Dr. A. L. Gray for the radiographic findings. It has been a matter of intense interest to watch the work of these two men. Almost invariably they have checked one another, not only as to the presence of stone, but as to its anatomical location as well.

The following case will illustrate the confusion of symptoms and the value of careful routine examination.

Case I. White; female; aged 36; married, mother of three children; previous history negative. About two years ago she had an attack of pain in her right side, not very severe. Since this time she has had numerous attacks, some slight, some severe and becoming more severe of late. The attacks seem to bear a definite relation to her menstrual periods, coming almost always with the beginning or termination of the menses and, of late, almost of monthly occurrence. The pain is very severe, beginning over the appendix and radiating down toward the right tube. At times it was also in the lumbar region. There was never a rise of temperature. The last attack occurred about ten days before coming to the hospital; and the diagnosis was recurrent appendicitis.

Vaginal examination revealed a badly lacerated cervix and perineum, and a retroverted uterus; the tubes and ovaries were normal. Microscopic examination revealed a few blood-cells and quite a number of cells from the kidney pelvis.

*Diagnosis.*—Stone in the pelvis of the kidney. A radiographic examination was then made, the report from it being a stone, the size of a cherry seed in the pelvis of the kidney.

Upon operation this was verified, and the stone removed through a small slit in the pelvis.

With the lacerated cervix, the retroverted uterus and the very definite relation between the menses and the attacks of pain; with the diagnosis of appendicitis, it would have been very easy to fall into error, do a section, suspend the uterus and remove the appendix. These procedures, however, would not have improved the symptoms from which she sought relief.

Case II. White; male; aged 32; clerk; gave a history of his first attacks of pain about three years previously. Up until the present year, he has had about 12 attacks. During the past year, there have been fifteen attacks, which are increasing in frequency and severity. The pain is in the right side, above and to the inner side of McBurney's point. It never radiates down the thigh or into the head of the penis. It has never been lumbar in character. It is so severe that two H. M. C. tablets have failed to relieve him. There has never been any elevation of temperature. There has never been any tenderness anywhere; never any macroscopic blood in the urine. With two attacks, there was a decided icteric tinge to the skin. The location of the pain, coupled with jaundice, strongly suggested gall-stones. Urinalysis showed blood- and kidney-epithelium. A radiograph revealed a stone in the kidney substance. On the operation, stone was removed from the cortico-medullary junction.

This case also illustrates how the pain and other physical signs may mislead, and emphasized the value of routine effort to eliminate the kidney.

Before closing, I wish to call attention to two other peculiar conditions, which I have not seen described, which bear a close relation to one another and seem based on faulty metabolism. I call attention to them because they seem to me definite clinical entities with a similar if not an identical origin. The one is an accompaniment of marked oxaluria, the other of a marked acidosis usually associated with pregnancy. By reporting a typical case of each, I can best describe their symptomatology.

Case 1. A white; female; aged 28; married, no children, while convalescent from an operation for a very bad case of pus-tubes, in which both tubes and the appendix were removed and free drainage used, was seized with a violent, right-kidney colic which required repeated doses of morphine to relieve. After recovering, I lost sight of her for a year, when she returned with an almost constant pain in her right side, beginning in the back and running down to the appendicular region. In addition to the usual pain, there was frequent violent paroxysms.

The tubes and the appendix had been eliminated. Urinalysis showed blood-cells and a very abundant deposit of oxalates. I felt confident that a radiograph would reveal stone. She was given an ounce of castor oil preliminary to taking the radiograph; and stated the next day that the oil had cured her. The plate was negative, and she was sure that no stone had passed. She has no attacks as long as the bowels are kept open and the oxalates held in solution by giving mineral acids. Constipation and faulty metabolism, as exhibited in the oxaluria, seem here a reasonable explanation of her condition. Why the pain should be unilateral I do not attempt to explain. Unfortunately, her interest in her case was of purely practical kind, stopping with the cessation of pain, so that I was unable to note the subsequent condition of the urine. I have seen a few other cases under this group, but none so typical.

Case II. This illustrates the second group. A white female; aged 28; married; had suffered several attacks of appendicitis in past years. When about six months pregnant, she was taken with a severe pain over the appendix accompanied by persistent vomiting, elevation of temperature, rapid pulse, and exquisite tenderness over McBurney's point. Muscular rigidity, tympany and lumbar tenderness were absent. The condition had existed several days without improvement. Vaginal examination revealed a normally pregnant uterus. Urinalysis showed abundant acetone and diacetic acid; otherwise, there was nothing significant.

She was given one drachm of sodium bicarbonate by mouth every hour, and saline by rectum, following which the urine became alkaline and all symptoms grew better. She was then put on a starchy diet, and the acetone and acetic acid quickly disappeared. The appendix was then removed. It bore evidence of former attacks, but its condition would hardly have warranted the rather severe symptoms occurring just prior to operation.

I have observed several cases with histories almost identical with this one, usually in pregnanet women, but time will not permit a further recitation.

To summarize: There is in addition to the acute inflammation of the kidney substance and its pelvis and the symptoms due to the presence of stone, a definite group of symptoms closely resembling those of acute appendicits. This condition seems to be due to faulty metabolism, evidenced, in the first instance, by oxaluria; and in the second, by urine loaded with acetone and diacetic acid.

Whether the pain is colonic or nephritic, I do not know. It certainly bears a close resemblance to the pain of appendicular or tubal inflammation. The absence of rigidity and tympany, and the primary local origin of the pain are the chief differential signs. Clearing the alimentary canal and the administration of a mineral acid, to hold the oxalates in solution, give relief in the one; while neutralization of the urine with alkalines and the administration of starchy foods correct the other.

In making a diagnosis of the second condition, one should exercise the greatest caution, for acidosis frequently accompanies intra-abdominal inflammation; and both acetone and diacetic acid are often found in these cases, especially when food has been withheld for any length of time. On the other hand, recognition of a marked acidosis is always of the highest importance whether it be associated with a true surgical condition or be but a signal of a purely medical one, for the administration of a general anesthetic in either instance by exciting the elaboration of more toxic substances and crippling the organs of elimination, may easily tip the already lightly balanced scale and cause a fatal termination.

I realize fully that acute inflammations of the right abdomen and especially of the appendix, are matters for early decision and radical action, and that delay and temporizing may cost a life. There are cases so evident, so clear, so urgent, that physical findings alone may be sufficient when laboratory accesories are absent or inconvenient. But there are many, many other cases less urgent in which the question of life and death is not uppermost. In these, at least, let us make routine vaginal examinations to exclude the tubes, and careful microscopic examinations of the urine, and if this be suggestive, a radiograph of the kidney and ureter, and thus exclude that most disquieting factor, the right kidney.

100 West Grace Street.

